# How Fencing Affects the Soil Quality and Plant Biomass in the Grassland of the Loess Plateau

**DOI:** 10.3390/ijerph14101117

**Published:** 2017-09-25

**Authors:** Quanchao Zeng, Yang Liu, Li Xiao, Yimei Huang

**Affiliations:** Key Laboratory of Plant Nutrition and the Agri-Environment in Northwest China, Ministry of Agriculture, College of Natural Resources and Environment, Northwest A&F University, Yangling 712100, China; quanchaozeng@umass.edu (Q.Z.); roshanlx@163.com (Y.L.); xiaoli047@126.com (L.X.)

**Keywords:** above-ground biomass, carbon stocks, grazing exclusion, plant diversity, succession time

## Abstract

Overgrazing is a severe problem in several regions in Northwestern China and has caused serious land degradation. Secondary natural succession plays an important role in the accumulation of soil carbon and nitrogen contents. Estimating the effects of grazing exclusion on soil quality and plant diversity will improve our understanding of the succession process after overgrazing and promote judicious management of degraded pastures. This experiment was designed to measure soil properties and plant diversity following an age chronosequence of grasslands (ages ranged from one year, 12 years, 20 years, and 30 years) in Northwestern China. The results showed that continuous fencing resulted in a considerable increase in plant coverage, plant biomass (above- and below-ground biomass), and plant diversity, which can directly or indirectly improve the accumulation of soil organic carbon and total nitrogen content. The plant coverage and the above- and below-ground biomass linearly increased along the succession time, whereas soil organic C and N contents showed a significant decline in the first 12 years and, subsequently, a significant increase. The increased plant biomass caused an increase in soil organic carbon and soil total nitrogen. These results suggested that soil restoration and plant cover were an incongruous process. Generally, soil restoration is a slow process and falls behind vegetation recovery after grazing exclusion. Although the accumulation of soil C and N stocks needed a long term, vegetation restoration was a considerable option for the degraded grassland due to the significant increase of plant biomass, diversity, and soil C and N stocks. Therefore, fencing with natural succession should be considered in the design of future degraded pastures.

## 1. Introduction

Overgrazing has accelerated soil erosion and severe degradation in arid and semiarid grasslands over recent decades [1,2,3] as it has significantly reduced plant cover [4], declined plant height and biomass [5], decreased plant richness [6], and reduced soil organic carbon stocks reviewed by Dlamini [7]. Therefore, relieving the serious degradation of grasslands is vital around the world [8,9].

The installation of fencing and vegetation restoration have been implemented to reduce the risks of serious soil erosion and reverse degradation trends [2,8,10]. For example, Wu et al. (2010) observed that a nine-year fencing significantly enhanced the vegetation cover and the above-ground biomass [10]. Deng et al. revealed that the plant height and the plant above biomass increased by 138% and 172% separately after fencing for 30 years in Chinese grasslands. The results from a meta-analysis on Chinese grasslands showed that the grazing exclusion had little effect on plant diversity recovery, but enhanced the soil carbon storage [11]. A short term grazing experiment showed that soil bulk density, soil organic carbon and above-ground net primary productivity were sensitive to grazing with different intensities [3]. These findings revealed that fencing enhanced the functioning of the grassland ecosystem.

Grazing exclusion through fencing is regarded as the most effective method available for ecological restoration on the Loess Plateau of China because of its complex terrain, extreme drought conditions, and severe soil erosion [2]. Although many studies have focused on overgrazing in different areas, the effects of the above- and below-ground biomass, the soil nutrients and their stocks need to be more specific. Therefore, this study was conducted to assess the variations in soil quality and plant diversity and their relationships across a restoration sequence (age changed from one year, 12 years, 20 years, and 30 years) on the grasslands in Northwestern China. The study was designed to enhance our understanding of the effect of grazing exclusions and restoration sequences on the functioning of grasslands. In addition, information regarding soil and plants will provide effective methods of managing the degenerated pastures.

Previous study in the Yunwu Mountain reserve showed that soil bacterial communities had a successional dynamic along a chronosequence [12]. Although most studies have focused on fencing, the association of the soil properties and plant diversity was not clear. Therefore, this study aimed to provide new insights into how vegetation succession affected soil quality in response to fencing along an age sequence in Northwest China. Thus, we addressed the following three hypotheses: (1) plant diversity and above- and below-ground biomass should be enhanced across the succession; (2) soil carbon and nitrogen stocks increased with the increase of age duration as plant biomass was enhanced; and (3) the relationships between plant diversity and soil quality were significantly related along the succession age. This study strengthens the current understanding of the restoration of soil C, N, and P sequestration and the relationships between soil and plant diversity in degraded grasslands on the Loess Plateau.

## 2. Materials and Methods

### 2.1. Experimental Site

**Figure 1:**
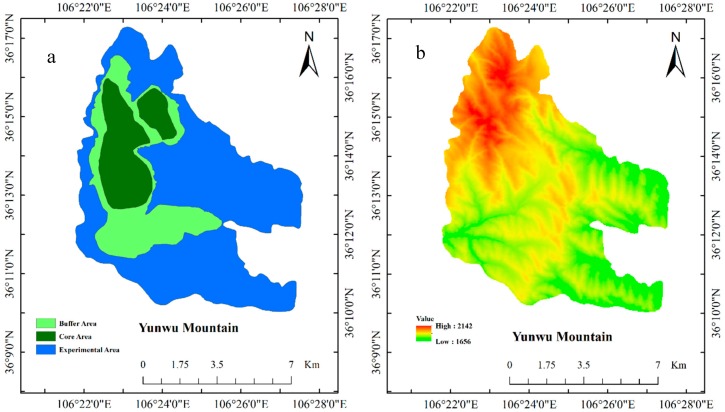
The studied area under different succession stages. (**a**) the setup of different functional areas; (**b**) the digital elevation model map.

The experimental site, the Yunwu Mountain Reserve, is located in Guyua City, Northwest China, and is a typical grassland with different successional ages after fencing. In this study, we chose four successional ages, including the one-year site, 12-year site, 20-year site, and 30-year site. Prior to fencing, the grassland was seriously degraded due to overgrazing. The soil type of the experimental site was entisols based on the American taxonomy (Soil Survey Staff, 1988) [13,14,15]. The grassland was dominated by the *Stipa bungeana*, *Thymus mongolicus*, and *Artemisia vestita* [12]. The climate of the experimental site is semi-arid temperate continental monsoon climate. The average means of the annual temperature and the mean annual precipitation are appropriately 8 °C and 371 mm (1991–2015), respectively. Approximately 70% of the total rainfall occurred between June and September [12]. The reserve includes three areas: the core area, buffer area, and experimental area, which have comparatively similar geographical patterns and climate (Figure 1). The dominant plant species are listed in Table 1.

### 2.2. Experimental Design and Measurements

Soil samples were obtained during mid-August 2014 when the biomass had reached its peak for the successional sites. For each successional age, we selected five sampling sites as replicates for a total of 20 sampling sites (4 ages × 5 replicates) (Figure 1). The dominant plant species differed in restoration time. Three plots (20 × 20 m) were established for each sampling site, i.e., in total there were 60 plots. These plots were randomly arranged in the field. Five 1-m^2^ quadrants were randomly established across each plot to represent heterogeneity. The biomass was estimated by collecting the above-ground biomass (AGB) and below-ground biomass (BGB, root) of all plants in one 1-m^2^ sampling quadrants in each plot. We dipped the 0–40 cm soil layer and sieved to obtain the roots (BGB). All roots were washed with distilled water, air-dried, and then oven-dried at 70 °C for at least 72 h or more to a constant weight before weighing. The total biomass (TB) was the sum of the AGB and BGB. TB, AGB and BGB were expressed as t/ha.

The number of plant species was used to estimate the species richness (*S*). The Shannon-Wiener Index was calculated using the Equation (1) [16,17]:

(1) $$H=-\overset{S}{\underset{i=1}{\sum }}\left(P_{i}lnP_{i}\right)$$

where *P_i_* is the relative abundance of each species; *S* is the total number of species; and *ln* is the log base-e. 

Soil samples were collected using seven soil cores in each plot. Soil core was obtained using a 5-cm diameter soil auger and pooled to a composite sample. The soil samples were obtained from 0 to 20 cm. The roots, stones, and animals were removed, air-dried, and finely ground through a 0.15-mm sieve for the C, N and P analysis.

Soil bulk density (BD) was measured using the undisturbed core method, using a 100 cm^3^ ring (three replicates in each sample site) [18]. The concentrations of soil total N (STN), soil organic carbon (SOC) and soil total nitrogen phosphorus (STP) were determined by the method described in the previous study [19]. The total SOC, STN, and STP stocks (t/ha) were calculated using Equation (2) [20,21]:

(2) $$Element_{stock}=element_{content}\times BD\times d$$

where *element_content_* is the soil organic carbon content (%), total nitrogen (%) and total phosphorus (%), respectively; *BD* is the soil bulk density (*BD*; g/cm^3^); and *d* is the soil depth (cm) of 20 cm.

### 2.3. Statistical Analysis

All the data in the present study were expressed with mean ± standard error. The one-way analysis of variance (ANOVA) was analyzed to investigate the effects of successional age on the soil properties studied and the plant characteristics. The Pearson correlations or linear regressions were used to determine the relationships between the nutrients in soil and plant characteristics. The principal component analysis (PCA) was conducted using Canoco 5.0 (Microcomputer Power, Ithaca, NY, USA) for Windows to test the relationships between soil C, N, and P content and plant characteristics. ANOVA and Pearson correlation analyses were conducted using the SPSS 20.0 software (SPSS, Inc., Chicago, IL, USA). The significance was at the 0.05 level. The figures were performed using Origin Pro 2016 (OriginLab Corporation, Northampton, MA, USA).

## 3. Results

### 3.1. Plant Characteristics’ Response to Restoration Time

**Figure 2:**
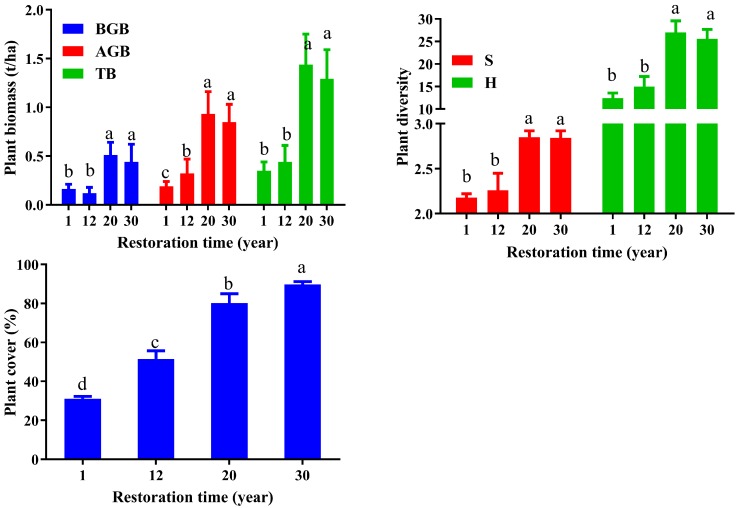
The characteristics of the vegetation across the succession (mean ± standard deviation, *n* = 9). AGB, above-ground biomass; BGB, below-ground biomass; TB, total biomass; *S*, species richness index; H, Shannon diversity index. Different letters indicate significant differences between vegetation sites (*p* < 0.05).

In this study, the plant diversity indices and plant biomasses showed some remarkable successional changes along a chronosequence (Figure 2), suggesting that fencing had a positive effect on plant biomass and plant diversity (*p* < 0.05). As expected, plant TB, AGB, and BGB significantly increased with successional age after fencing. Plant AGB increased from 0.19 to 0.85 t/ha, with the highest result obtained in the 20-year site (0.93 t/ha), but there was no significant difference between the 20-year and the 30-year site (*p* > 0.05). Plant BGB increased in the first 20 years, then decreased with fencing time, and reached a peak at the 20-year site (0.51 t/ha). TB showed a similar trend with BGB, with a significant lower result in the one-year and 12-year sites than the 20-year and 30-year sites (*p* < 0.05). Plant cover significantly linearly increased from 31% to 90% (*p* < 0.05), reaching a peak at the 30-year site (90%). There was a significant difference across all succession stages (*p* < 0.05). The H index could indicate the diversity of the plant community in the grassland with a significant higher H index in the 20-year and the 30-year sites than those in the one-year and 12-year sites (*p* < 0.05). The *S* index also showed similar varied trends with the H and Shannon indices, with a range from 13 to 26. The H index and *S* index remained stable after a 20-year succession, whereas plant cover and AGB continued to increase. H and *S* showed no significant difference between the one-year and the 12-year sites, as well as between the 20-year and the 30-year sites (*p* > 0.05).

### 3.2. Soil Properties Response to Restoration Time

**Figure 3:**
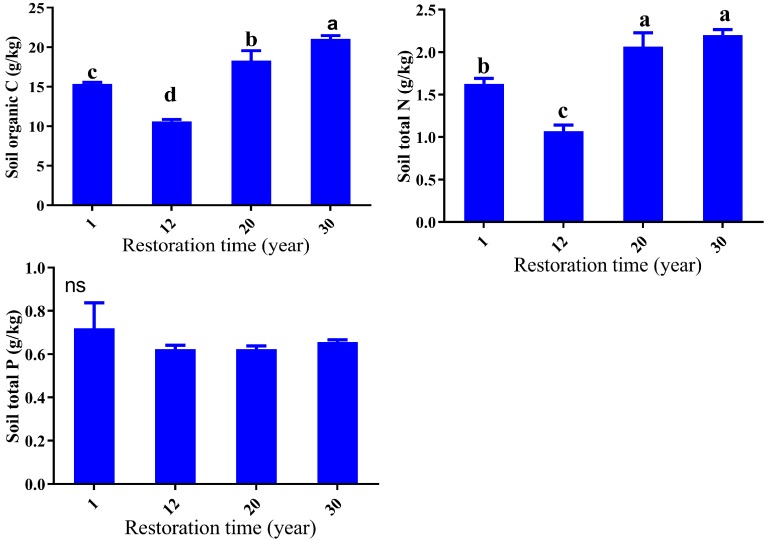
Soil organic carbon (SOC), soil total nitrogen (STN), and soil total phosphorus (STP) across different succession stages. Different letters indicate significant differences between vegetation sites (*p* < 0.05), ns indicated that there was no significant difference for STP across the succession.

The vegetation restoration age had significant effects on the nutrient contents in soil. The concentration of SOC showed a significant decline in the first 12 years and then significantly increased with a peak at the 30-year site (*p* < 0.05) (Figure 3). The pattern of STN concentration was similar to that of SOC concentration (Figure 3). STN contents ranged between 1.07–2.20 g/kg and significantly differed across different succession stages (*p* < 0.05). In the first 12 years, STN contents decreased by 34%, then increased 51% after 30 years. The range of STP concentration was rather narrow (0.62–0.72 g/kg), with no significant difference among the restoration times (*p* > 0.05) (Figure 3).

### 3.3. Soil Bulk Density, Carbon, Nitrogen, and Phosporus Stocks Response to Restoration Time

**Figure 4:**
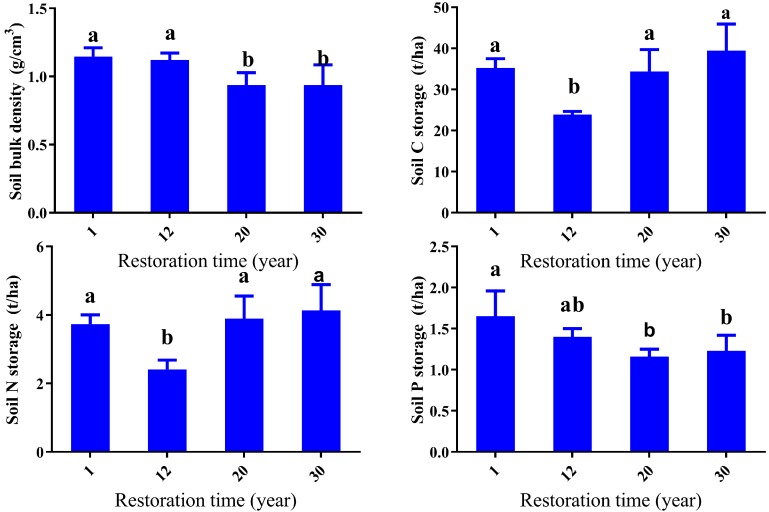
Soil bulk density, carbon, nitrogen, and phosphorus stocks across different succession stages. Different letters indicate significant differences between vegetation sites (*p* < 0.05).

Soil bulk density ranged from 0.94 to 1.15 g·cm^−3^ and was significantly lower at the 20-year and 30-year sites (*p* < 0.05) (Figure 4). Bulk density decreased after fencing with significant differences between the younger succession stages (one-year and 12-year sites) and the older succession stages (20-year and 30-year sites) (*p* < 0.05). There were no significant differences between the one-year and the 12-year sites, and the 20-year and the 30-year sites (*p* > 0.05) (Figure 4).

SOC stocks ranged between 24–40 t/ha and showed a similar pattern as the SOC contents, but were less explicit (Figure 4). Soil N stocks showed a similar trend with SOC stocks. Soil N stocks ranged from 2.4 to 4.1 t/ha, with the lowest one for the 12-year site and highest one for the 30-year site (Figure 4). There was no significant difference for the SOC stocks and TN stocks between the 20-year and the 30-year sites, indicating that after a 20-year restoration, SOC stocks and TN stocks remained stable.

### 3.4. The Relations between Soil Properties Studied and Plant Biomass across the Succession

**Figure 5:**
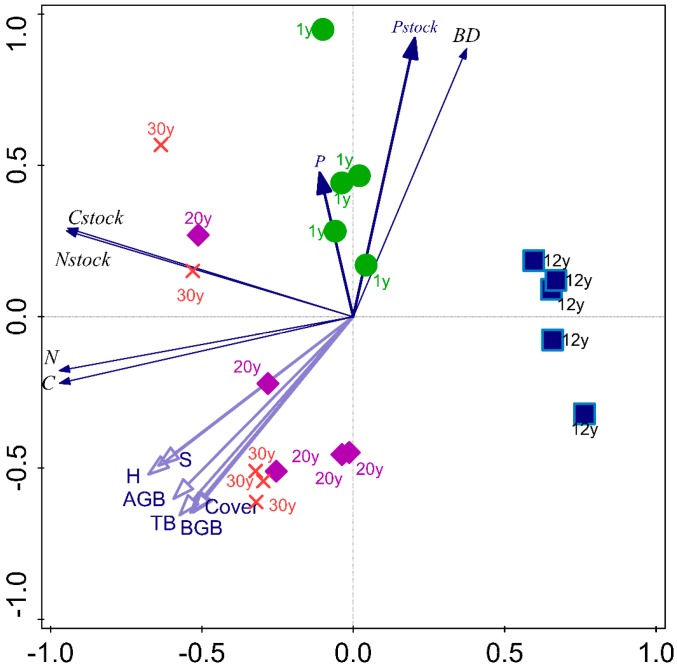
The relationships between soil C, N and P contents and stocks and plant characteristics. *S*, species richness; H, Shannon wiener index; BD, soil bulk density; SOC, soil organic carbon; TN, soil total nitrogen; TP, soil total phosphorus.

**Figure 6:**
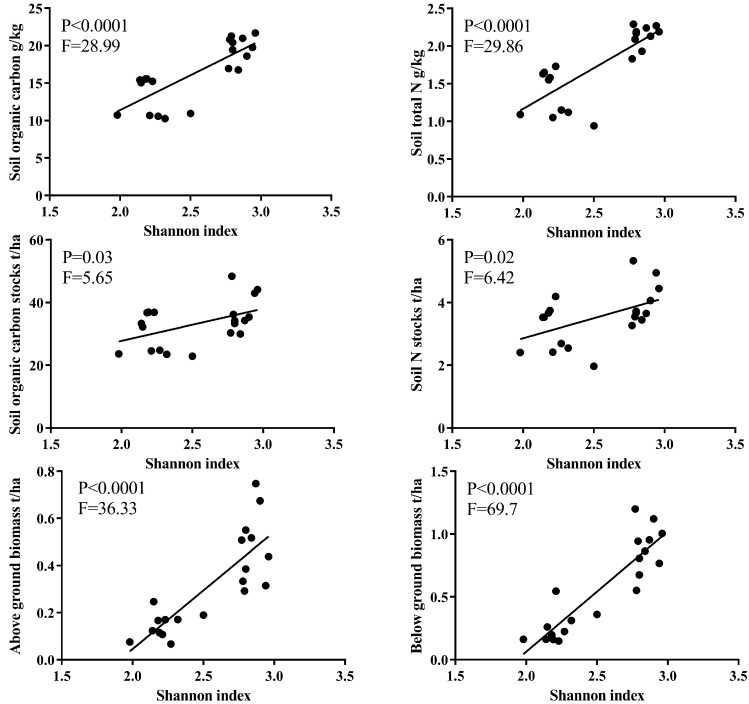
The regression between the Shannon index and SOC contents, TN contents, SOC stocks, TN stocks, AGB, and BGB.

Table 2 and Figure 5 show the relationships between soil properties and plant above- and below-ground biomass. BGB was significantly related to SOC contents, TN contents and P stocks (*p* < 0.05) (Table 2). There were no significant relationships between the AGB and SOC stocks and TN stocks. As expected, plant coverage and plant diversity significantly correlated with SOC and TN stocks (*p* < 0.05) (Figure 6). These results showed the plant biomass and diversity had significant effects on the accumulation of SOC and TN. The Shannon index was significantly linearly correlated with SOC contents (*p* < 0.0001, *F* = 28.99), TN contents (*p* < 0.0001, *F* = 29.86), SOC stocks (*p* = 0.03, *F* = 5.65), TN stocks (*p* = 0.02, *F* = 6.42), AGB (*p* < 0.0001, *F* = 36.33), and BGB (*p* < 0.0001, *F* = 69.7) (Figure 6).

## 4. Discussion

The results showed that the vegetation succession with fencing significantly influenced most of the studied soil properties. A significant increase occurred in the 20-year site (*p* < 0.05). The lowest SOC content and stock were found for the 12-year site, which can be explained by the fast growth of plants during the early successional period. The restoration for soil nutrients is a slow process and it needs more time. Significantly higher SOC contents were found for the 20-year site (*p* < 0.05), which was confirmed by the previous study [22]. In their study, soil organic matter increased by 18% after the conversion from farmland to natural grassland [22]. In the present study, SOC increased by 19% after 20-year fencing. SOC stocks were relatively high and varied from 24 to 39 t/ha. Similar changes were observed in the humid Mediterranean landscape with a range from 26 to 58 t/ha along a 50-year succession [23]. From 12 years to 30 years, SOC accumulated at the rate of 0.87 t/ha per year, which was significantly higher than the mean rate of 0.62 t/ha per year in the pastures of Central America [24], and in the establishment of perennial vegetation on croplands (0.37 t/ha per year) [25], and the establishment of *Medicago* in China (0.27 t/ha per year) [26].

Vegetation succession also significantly influenced the soil TN (*p* < 0.05). Similar to the results found for SOC, the lowest TN contents were found at the 12-year site. Significantly higher contents were found for the 20-year and 30-year sites (*p* < 0.05), which was in accordance with previous results [22,23]. In the first 12 years, the plants without any disturb grew rapidly and needed many nutrients which resulted in the decline of soil TN contents and SOC contents. However, the plant cover and plant above-ground biomass continuously increased, which confirmed the decline of soil SOC and TN contents. The opposite varied trend indicated that, in the early succession stage, soil TN and SOC stocks reduced along with the increase of plant coverage and biomass. These results suggested that the SOC and TN accumulations lagged behind the plant cover and biomass.

Along with the succession, the soil bulk density decreased from 1.15 g/cm^3^ to 0.94 g/cm^3^. Similar variations were reported in the Loess Plateau [2] and the Mediterranean area [23]. A significantly higher bulk density was found in one-year and 12-year sites due to overgrazing before fencing. This was confirmed by the case that overgrazing had negative effects on soil structure [27,28]. The improvement of soil structure needs a long time. During the vegetation restoration process, the decomposition of litters and roots caused higher organic matter. The accumulation of soil organic carbon and total nitrogen was one explanation for the reduction of soil bulk density [29].

Our study also demonstrated that plant biomass had a significant effect on the soil properties studied (Table 2 and Figure 5). Long-term fencing enhanced plant biomass and plant diversity, which was in accordance with studies in [2,8,30]. Many previous studies have reported that plant coverage, above-ground biomass, and below-ground biomass were the main drivers of the accumulation of SOC [31]. Higher AGB caused higher litters to be returned to the soil via litter decomposition, leading to the accumulation of soil organic matter [32]. The significant relationships between SOC and the above- and below-ground biomass confirmed the effects of plant biomass on the soil properties. Fine root dynamics is a main driver of soil carbon stocks [33], particularly in arid grasslands. As roots are conduits for nutrients from the soil to the plants, they play an important role in resisting variations in environmental constraints [34,35]. Root biomass strongly affected SOC contents and stocks as reported in previous studies [33,36]. Rasse et al. [36] reported that root activities were the main protection mechanisms for SOC. Furthermore, SOC increased with plant productivity which, in turn, could give positive feedback to plant productivity via plant litter decomposition [37].

Plant diversity strongly influences ecosystem functions and services, such as soil carbon and nitrogen storage. Plant species richness and the Shannon index enhanced SOC and TN stocks, which have been confirmed by previous studies [37,38]. In this study, the significantly positive effects of the Shannon index on AGB, BGB, and soil C stocks suggested that the plant Shannon index promoted C inputs into the soil (Figure 6). The positive effect of plant diversity on SOC stocks could have been caused by the higher input of plant production and decreased the C losses [2,37].

On the other hand, fencing can also enhance the ability to resist soil erosion via plant coverage [39]. The increase in plant biomass at degraded areas is associated with higher vegetation coverage and a stronger capacity to control soil erosion in semi-arid regions [40]. This may be another explanation for the accumulation of SOC and STN across the succession. Although this research did not detect soil aggregate stability which could reflect the ability of resistance to soil erosion, plant coverage and SOC could indirectly reflect soil aggregate ability [41,42,43,44]. SOC contents were positively related with soil aggregate ability [45]. With higher plant diversity and plant coverage, soil erosion can be reduced and subsequently prevent the loss of soil organic carbon and nitrogen via leaching or washing.

## 5. Conclusions

Overgrazing is one of the main causes of land degradation in the semi-arid grasslands of Northwestern China. Continuous fencing resulted in a considerable increase in plant coverage and biomass, which enhanced the resistance to the loss of soil organic C and N and subsequently caused the accumulation of soil organic carbon and total nitrogen. These results indicated that vegetation restoration with fencing on degraded grasslands has great potential to improve soil fertility, sequester soil organic carbon and nitrogen, and improve plant above- and below-ground biomass. Soil and plant restoration was an incongruous process. Soil restoration was a slow process and fell behind vegetation restoration after long-term fencing. Compared with the above-ground biomass, the below-ground biomass was the main driver behind the accumulation of SOC and TN contents. Therefore, fencing with natural restoration should be considered in the management of degraded pastures in the Loess Plateau.

## Figures and Tables

**Table 1:** Descriptions of the soil sample sites under different restoration ages.

Ages	Altitude/m	Longitude	Latitude	Dominant Species
1a	1762–1774	106°24′15.61′′	36°10′2.01′′	*Stipa bungeana*; *Artemisia scoparia*; *Potentilla acaulis*; *Heteropappus altaicus*
12a	1778–1795	106°24′48.46′′	36°10′28.05′′	*Thymus mongolicus*; *Leymus secalinus*; *Artemisia scoparia*; *Stipa bungeana*
20a	2077–2104	106°23′20.87′′	36°16′32.46′′	*Heteropappus altaicus*; *Cyperaceae*; *Thymus mongolicus*; *Artemisia vestita*
30a	2083–2115	106°23′15.76′′	36°16′2.14′′	*Artemisia vestita*; *Stipa przewalskii*; *Heteropappus altaicus*

**Table 2:** The Pearson relationships between soil properties and plant characteristics.

Soil Properties	BGB	AGB	TB	Cover	*S*	H
BD	−0.786 **	−0.776 **	−0.811 **	−0.752 **	−0.656 **	−0.696 **
SOC	0.697 **	0.669 **	0.708 **	0.697 **	0.741 **	0.785 **
TN	0.736 **	0.661 **	0.717 **	0.685 **	0.758 **	0.790 **
TP	−0.16	−0.275	−0.241	−0.341	−0.267	−0.252
C-stocks	0.318	0.289	0.314	0.331	0.467 *	0.489 *
N-stocks	0.382	0.307	0.349	0.346	0.506 *	0.513 *
P-stocks	−0.632 **	−0.696 **	−0.698 **	−0.726 **	−0.609 **	−0.631 **

* indicates significant relationships at the level of 0.05; ** indicates significant relationships at the level of 0.01. AGB, the above-ground biomass; BGB, the below-ground biomass; TB, total biomass; SOC, soil organic carbon; TN, soil total nitrogen; *S*, species richness index; H, Shannon-Wiener index.
